# Differential Mechanisms of Myocardial Conduction Slowing by Adipose Tissue‐Derived Stromal Cells Derived from Different Species

**DOI:** 10.5966/sctm.2015-0415

**Published:** 2016-08-02

**Authors:** Judith N. ten Sande, Nicoline W. Smit, Mojtaba Parvizi, Shirley C.M. van Amersfoorth, Josée A. Plantinga, Pascal F.H.M. van Dessel, Jacques M.T. de Bakker, Marco C. Harmsen, Ruben Coronel

**Affiliations:** ^1^ Heart Center, Department of Clinical and Experimental Cardiology, Academic Medical Center, University of Amsterdam, Amsterdam, The Netherlands; ^2^ Interuniversity Cardiology Institute of the Netherlands, Netherlands Heart Institute, Utrecht, The Netherlands; ^3^ Department of Pathology and Medical Biology, University Medical Center Groningen, University of Groningen, The Netherlands; ^4^ L'Institut de Rythmologie et de Modélisation Cardiaque, Université Bordeaux, Segalen, Bordeaux, France

**Keywords:** Adipose stromal cells, Cardiomyocytes, Electrophysiology, Conduction slowing, Paracrine

## Abstract

Stem cell therapy is a promising therapeutic option to treat patients after myocardial infarction. However, the intramyocardial administration of large amounts of stem cells might generate a proarrhythmic substrate. Proarrhythmic effects can be explained by electrotonic and/or paracrine mechanisms. The narrow therapeutic time window for cell therapy and the presence of comorbidities limit the application of autologous cell therapy. The use of allogeneic or xenogeneic stem cells is a potential alternative to autologous cells, but differences in the proarrhythmic effects of adipose‐derived stromal cells (ADSCs) across species are unknown. Using microelectrode arrays and microelectrode recordings, we obtained local unipolar electrograms and action potentials from monolayers of neonatal rat ventricular myocytes (NRVMs) that were cocultured with rat, human, or pig ADSCs (rADSCs, hADSCs, pADSCs, respectively). Monolayers of NRVMs were cultured in the respective conditioned medium to investigate paracrine effects. We observed significant conduction slowing in all cardiomyocyte cultures containing ADSCs, independent of species used (*p* < .01). All cocultures were depolarized compared with controls (*p* < .01). Only conditioned medium taken from cocultures with pADSCs and applied to NRVM monolayers demonstrated similar electrophysiological changes as the corresponding cocultures. We have shown that independent of species used, ADSCs cause conduction slowing in monolayers of NRVMs. In addition, pADSCs exert conduction slowing mainly by a paracrine effect, whereas the influence on conduction by hADSCs and rADSCs is preferentially by electrotonic interaction. Stem Cells Translational Medicine *2017;6:22–30*

**Significance Statement**

Cell‐based therapy is a promising option to treat patients after myocardial infarction. Although cell‐based therapy may help replace infarcted heart tissue by functional tissue, it has some limitations. First, it may cause life‐threatening arrhythmias. Slow conduction facilitates arrhythmias induction. Second, cells derived from and administered to the same patients may be affected by age and disease. Therefore, cells from other patients or other species may be used. This study shows that application of stromal cells caused conduction slowing in cardiomyocyte monolayers, irrespective of the specific origin of the cells, but that the conduction slowing is conferred through soluble factors or through coupling between fat‐derived cells and cardiac myocytes in a species‐dependent manner.

## Introduction

Up to one third of the patients with myocardial infarction develop heart failure despite improvements in reperfusion therapy 1. Stem cell‐based therapy has been suggested as a promising therapeutic modality to improve cardiac function in these patients 2 3 4. However, there are concerns for the potential proarrhythmic effects of stem cell therapy 5 6 7. One proposed mechanism for the proarrhythmic potential is the formation of electrotonic interaction between cardiomyocytes and stem cells, allowing interaction between the interiors of the two cells 8. The membrane potential of mesenchymal stem cells is approximately −35 mV 9, 10. As a consequence, electrotonic coupling between a stem cell and a ventricular myocyte is expected to cause depolarization and a change in the action potential morphology of the myocytes. This may result in conduction slowing, conduction heterogeneity, and unidirectional conduction block, together facilitating re‐entrant arrhythmias 11, 12. A second suggested pathway is the involvement of paracrine factors that can directly or indirectly (paracrine cross‐talk) influence cardiomyocyte and/or stem cell function 7.

Another drawback of cell‐based therapies concerns the availability of stem cells. Autologous stem cells, such as mesenchymal stem cells from bone marrow, are not only rare but also difficult to obtain and expand to the large number required for treatment. Multipotent cells, such as adipose tissue‐derived stromal cells (ADSCs) are, however, highly abundant in lipo‐aspirates, which are easy to obtain from healthy individuals. ADSCs are not only abundantly present but also a source of multipotent cells capable of differentiating along multiple lineage pathways with few immunological effects 13, 14. In addition, ADSCs secrete a wide variety of factors known to stimulate angiogenesis 15 and neovascularization 16, making them clinically relevant for possible cell‐based therapies; their use is favored to date. However, the function of autologous stem cells can deteriorate because of age and risk factors, such as hyperglycemia and hyperlipidemia, which are present in the elderly population, in whom myocardial infarctions are most prevalent 17, 18. Current studies that describe the safety and efficacy of allogeneic stem cells indicate that these can be used as an “off‐the‐shelf” alternative for autologous stem cells 19, 20. In addition, xenogeneic stem cells are considered an alternative to autologous stem cell administration and have been described frequently 21 22 23 24. The potential difference in the proarrhythmic effects of adipose‐derived stromal cells across species is unknown.

In this in vitro study, we specifically address the potential adverse electrophysiological effects that different adipose tissue‐derived stromal cells have on a confluent layer of neonatal rat ventricular cardiomyocytes (NRVMs). We specifically studied the different (allogeneic and xenogeneic) species sources of ADSCs: namely the rat, human, and pig.

## Materials and Methods

A detailed description of the methods can be found in the supplemental online data.

### Isolation and Culturing of Neonatal Rat Ventricular Myocytes

All animal experiments were approved by the local Animal Experiments Committee (Academic Medical Center, University of Amsterdam and University Medical Center Groningen, University of Groningen, The Netherlands) and carried out in accordance with national and institutional guidelines.

Briefly, hearts were explanted from 1‐ to 2‐day‐old Wistar rats. Ventricles were dissected into pieces and dissociated with trypsin (Becton Dickinson BV, Breda, The Netherlands, http://www.bd.com) and collagenase (230 units/mg; Worthington Biochemical Corp., Vollenhove, The Netherlands, http://www.worthington-biochem.com). Cells were preplated to minimize fibroblast contamination. The remaining myocardial cells were plated on fibronectin (BD Biosciences) coated multielectrode arrays (MEAs; Multi Channel Systems MCS GmbH, Reutlingen, Germany, http://www.multichannelsystems.com/) at a density of 1.4 × 10^5^ cells per cm^2^.

### Isolation and Culture of Adipose Tissue‐Derived Stromal Cells

ADSCs were isolated and cultured as described previously 25. Inguinal rat fat (male, Wistar, 7–8 months), porcine subcutaneous abdominal fat (male, 3–4 months [ provided by the Department of Experimental Surgery of the Academic Medical Center]), and human subcutaneous abdominal fat (donated by healthy patients with body mass index <30 kg/m^2^; Bergman Clinics, The Netherlands) were used. Tissue was minced and washed extensively with phosphate‐buffered saline, before being subjected to dissociation steps with collagenase (Roche Diagnostics, Mannheim, Germany, http://www.roche.com). The obtained stromal vascular fraction was then incubated with erythrocyte lysis buffer; after this, the cells were seeded at a density of 4 × 10^4^ cells per cm^2^, and ADSCs were propagated at a 1:2 ratio and used from passage 3 onward. Cells were referred to rat ADSCs (rADSCs), human ADSCs (hADSCs), or pig ADSCs (pADSCs).

The use of liposuction material as source of ADSCs was approved by of the local ethics committee of the University Medical Center Groningen because it was considered anonymized waste material. Yet, for each of these anonymous donations the clients gave their consent after information.

### Experimental Conditions

To investigate effects of ADSCs, cocultures of NRVM and ADSC were prepared. Four days after seeding NRVMs, ADSCs were added to the monolayers at a ratio of 1:1, and 2 days later electrophysiological measurements were performed.

To assess paracrine effects, conditioned medium (Cme) was collected from monolayers of NRVMs (Cme NRVM), cocultures (Cme NRVMs:ADSCs), and confluent cultures of ADSCs (Cme ADSC). Medium was also collected from cocultures with transwell inserts: This medium was called Cme transwell ADSC. In transwell experiments, NRVMs and ADSCs are cultured together without making direct contact. Cme was filtered (0.22 µm) before being added to monolayers of NRVMs only on day 4 of culture and 2 days before measurements.

### Electrical Mapping and Microelectrode Measurements

Electrophysiological parameters were determined by mapping the electrical activity of the monolayers. MEAs harbored 60 electrodes terminals, aligned in an 8 × 8 matrix with terminals in the core portion of the MEA ( supplemental online Fig. 1). Cultures were stimulated by using a bipolar extracellular stimulus electrode (twice diastolic stimulation threshold, 1‐ or 2‐millisecond pulse width). Conduction velocity (CV) and conduction heterogeneity were determined from the unipolar electrograms recorded. For each experiment, two monolayers of NRVMs served as control. Values obtained under different conditions were compared with the values of control monolayers of the same isolation. Resting membrane potential (RMP) and upstroke velocity of an action potential were determined from action potentials recorded during microelectrode measurements.

### Immunostainings

Cells plated in 12‐well plates used for immunofluorescence were cultured under the same conditions as cells on MEAs. Briefly, after fixation in 4% paraformaldehyde cells were permeabilized and blocked before being stained with primary and secondary antibodies. Examination was performed by Leica SPE confocal laser scanning and Leica Application Suite Advanced Fluorescence software (Leica Microsystems, Buffalo Grove, IL, http://www.leica-microsystems.com). Immunofluorescence images were analyzed using ImageJ software, version 1.50i (National Institutes of Health, Bethesda, MD, https://imagej.nih.gov/ij).

### Statistical Analysis

Continuous and normally distributed variables are presented as mean ± SD (unless otherwise mentioned) and were compared by using an independent *t* test. For more than two groups, a one‐way analysis of variance was performed with the Bonferroni correction as a post hoc analysis. In case of a skewed distribution, data are presented as median with the interquartile range and tested with the Mann‐Whitney test; in case of more than two groups, a Kruskal‐Wallis analysis was performed with post hoc analysis using the Dunn test. A *p* value of <.05 was considered to indicate statistically significant differences. All graphs were made by using GraphPad Prism software, version 5 (GraphPad Software, La Jolla, CA, http://www.graphpad.com/).

## Results

### Effects of Coculturing ADSCs With NRVMs

**Figure 1:**
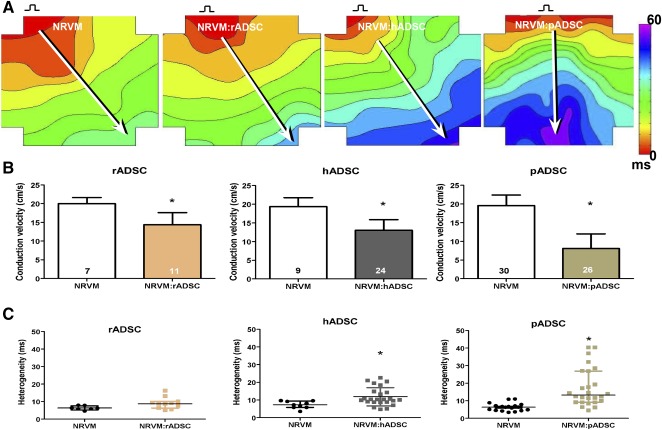
Effect of ADSCs on conduction velocity and heterogeneity in monolayers of NRVMs. **(A):** Activation map of a monolayer of NRVMs, a monolayer cultured with rADSCs, a monolayer with hADSCs, and a monolayer with pADSCs. Conduction velocity is determined along white arrows perpendicular to isochronal lines. **(B):** Conduction velocity of controls and different cocultures (mean ± SD). **(C):** Conduction heterogeneity. ∗, *p* < .001 compared with the monolayers of NRVM (median with IQR). Abbreviations: ADSC, adipose cell‐derived stromal cell; hADSC, human adipose cell‐derived stromal cell; IQR, interquartile; ms, millisecond; NRVM, neonatal rat ventricular myocyte; pADSC, pig adipose cell‐derived stromal cell; rADSC, rat adipose cell‐derived stromal cell.

Monolayers of NRVMs cocultured with rADSCs demonstrated conduction slowing compared with monolayers of NRVMs only (Fig. 1A). On average, conduction velocity was 14.4 ± 3.2 cm/second in monolayers of NRVM, cocultured with rADSCs, compared with 20.0 ± 1.6 cm/second in control monolayers (*p* < .001, Fig. 1B). Similar to rADSCs, monolayers that were cocultured with hADSCs (13.0 ± 2.8 cm/second) or pADSCs (8.0 ± 3.9 cm/second) also demonstrated significant conduction slowing compared with their respective controls (19.3 ± 2.4 and 20.2 ± 2.8 cm/second, respectively; *p* < .001, Fig. 1A, 1B).

Conduction heterogeneity in monolayers of NRVM cocultured with rADSCs demonstrated a trend to be higher compared with control monolayers (8.75 [interquartile (IQR), 3.8] vs. 6.2 [IQR, 1.95] milliseconds; *p* = .056, Fig. 1C). Heterogeneity in cocultures with hADSCs was on average higher than in control monolayers (10.3 [5.9] vs. 7.2 [5.1] milliseconds; *p* < .01, Fig. 1C). Monolayers cocultured with pADSCs (13.3 [17.7] milliseconds) also demonstrated a significant increase in conduction heterogeneity compared with monolayers of NRVMs only (6.4 [2.9] milliseconds; *p* < .001, Fig. 1C).

### Effects of Conditioned Medium of NRVM:ADSC

**Figure 2:**
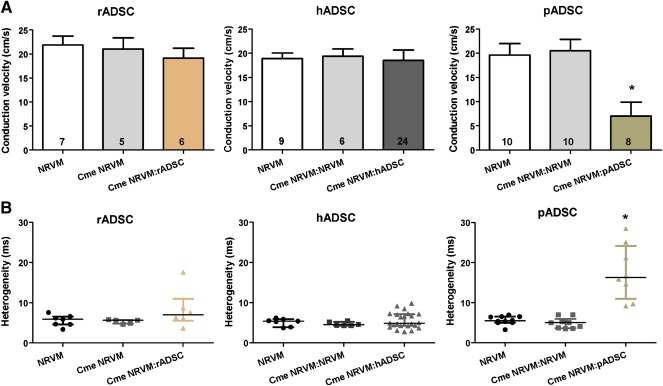
Effect of Cme ADSC:NRVM on conduction velocity and heterogeneity in monolayers of NRVM. Effects on conduction velocity (mean ± SD) **(A)** and conduction heterogeneity (median with interquartile) **(B)** in monolayers of NRVM cultured in the Cme obtained from the different cocultures. ∗, *p* < .01 compared with control monolayers and monolayers of NRVM cultured in Cme NRVM. Abbreviations: Cme, conditioned medium; hADSC, human adipose cell‐derived stromal cell; NRVM, neonatal rat ventricular myocyte; pADSC, pig adipose cell‐derived stromal cell; rADSC, rat adipose cell‐derived stromal cell.

To determine the mechanisms behind the conduction slowing, we cultured monolayers of NRVMs in Cme obtained from the various cocultures. Conduction velocity in NRVM monolayers cultured in Cme of the NRVM:rADSC cocultures was not different from conduction velocity (19.2 ± 2.0 cm/second) or conduction heterogeneity (7.0 [5.4] milliseconds) in control monolayers (21.8 ± 1.8 cm/second and 5.9 [1.9] milliseconds; *p* = n.s., Fig. 2A, 2B). Conduction velocity in NRVM monolayers cultured with Cme of NRVM:hADSC cocultures was also not affected compared with controls (18.5 ± 2.2 vs. 19.0 ± 1.2 cm/second; *p* = n.s., Fig. 2A). Conduction heterogeneity was not affected when NRVM monolayers were cultured in Cme NRVM:hADSC (4.9 [2.0] vs. 5.3 [1.9] milliseconds; *p* = n.s., Fig. 2B). In contrast, Cme NRVM:pADSC slowed conduction velocity significantly compared with control monolayers (7.0 ± 2.9 vs. 19.6 ± 2.4 cm/second; *p* < .001, Fig. 2A). Conduction heterogeneity was also significantly increased by Cme NRVM:pADSC compared with control monolayers (16.3 [13.2] vs. 5.5 [1.5] milliseconds; *p* < .001, Fig. 2B). Cme NRVM served as control for the conditioned medium conditions and did not differ from control monolayers in any of the groups (Fig. 2A, 2B). The CV or the heterogeneity in monolayers cocultured with pADSCs was not significantly different from the CV or the heterogeneity in monolayers of NRVMs cultured in Cme NRVM:pADSC (compare Fig. 1B, 1C vs. Fig. 2A, 2B).

**Figure 3:**
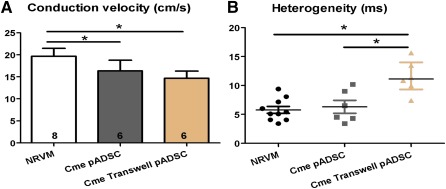
Effect of Cme pADSC transwell and Cme pADSC on conduction velocity and heterogeneity in monolayers of NRVM. Effects on conduction velocity (mean ± SD) **(A)** and conduction heterogeneity (median with interquartile) **(B)** in monolayers of NRVM cultured in Cme obtained from the transwell cocultures and pADSC culture. ∗, *p* < .05. Abbreviations: Cme, conditioned medium; NRVM, neonatal rat ventricular myocyte; pADSC, pig adipose cell‐derived stromal cell.

Conditioned medium of the cocultures NRVM:pADSC affected conduction properties of NRVM monolayers. To distinguish whether this effect is attributed to soluble factors of pADSCs or whether there is an interaction (cross‐talk and/or electrotonic connections) between pADSCs and NRVMs, we further explored the effects of Cme pADSC and Cme transwell pADSCs. NRVM monolayers cultured in Cme pADSC and Cme transwell pADSCs both demonstrated significantly lower conduction velocities compared with controls (16.3 ± 2.4 and 14.6 ± 1.6 vs. 19.6 ± 1.8 cm/second, respectively; *p* < .05, Fig. 3A). Conduction heterogeneity was affected only by Cme transwell pADSC (11.1 [4.9] vs. 5.7 [3.8] milliseconds; *p* < .05, Fig. 3B). Conditioned medium obtained from only hADSCs and rADSCs did not affect conduction velocity or the heterogeneity of NRVM monolayers ( supplemental online Fig. 2). In contrast to when monolayers of NRVM were incubated for 48 hours with Cme NRVM:pADSC, application immediately prior to electrical mapping of Cme NRVM:pADSC did not have an effect (results not shown).

### Microelectrode Measurements

**Figure 4:**
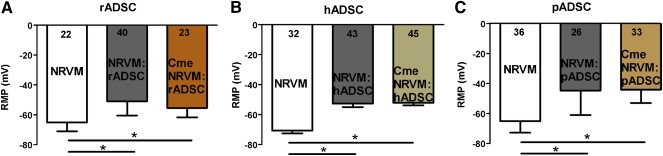
Effects of adipose cell‐derived stromal cells and Cme on membrane potential. The effects of coculturing rADSC **(A)**, hADSC **(B),** and pADSC **(C)** together with NRVM and the effects of Cme on resting membrane potential (mean ± SD). ∗, *p* < .001 compared with control monolayers (*N* = impalements). Abbreviations: Cme, conditioned medium; hADSC, human adipose cell‐derived stromal cell; NRVM, neonatal rat ventricular myocyte; pADSC, pig adipose cell‐derived stromal cell; rADSC, rat adipose cell‐derived stromal cell.

Microelectrode measurements were performed to study whether the observed conduction slowing could be explained by depolarization. As expected, monolayers of NRVM cocultured with rADSCs, hADSCs, and pADSCs were depolarized compared with control monolayers (RMP, −50.95 ± 9.45 vs. −65.06 ± 5.98 mV, −52.6 ± 15.2 vs. −71.2 ± 13.1 mV and −44.7 ± 16.2 vs. −66.0 ± 7.9 mV, respectively; *p* < .01, Fig. 4). Although monolayers cultured in Cme NRVM:rADSC and Cme NRVM:hADSC demonstrated no effect on conduction velocity, these monolayers were depolarized compared with controls (−55.4 ± 6.2 vs. −65.1 ± 6.0 mV and −52.1 ± 12.8 vs. −71.2 ± 13.1 mV, respectively; *p* < .01). Cme NRVM:pADSC elicited heterogeneous conducting slowing, and these cultures were also depolarized compared with controls (−44.0 ± 9.0 vs. −66.0 ±7.9 mV; *p* < .01, Fig. 4). Depolarization in NRVM monolayers induced by Cme NRVM:pADSC was significantly greater compared to the depolarization induced by Cme NRVM:rADSC (*p* < .01) and Cme NRVM:hADSC (*p* < .01).

### Relationship Between RMP and Conduction Velocity

**Figure 5:**
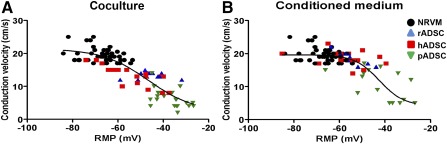
Relationship between RMP and conduction velocity. The relationship between RMP and CV in coculture situations **(A)** and monolayers cultured in conditioned medium **(B)** from the respective cocultures. Abbreviations: hADSC, human adipose cell‐derived stromal cell; NRVM, neonatal rat ventricular myocyte; pADSC, pig adipose cell‐derived stromal cell; rADSC, rat adipose cell‐derived stromal cell; RMP, resting membrane potential.

A theoretical sigmoid relation exists between RMP and CV 26, 27. We studied whether the relation between local RMP and conduction velocity was maintained in cocultures and after culturing in the presence of Cme. Figure 5A shows the relation between RMP and CV in the three different cocultures as well as in the pooled control monolayers (NRVM). In a similar fashion, Figure 5B shows the combined data of monolayers subjected to Cme of the various species and their corresponding pooled controls. In both panels, a sigmoid function is fitted through the combined data points (black lines). Because the average data do not appear to deviate from the theoretical sigmoid function, the figures show that the degree of depolarization of each monolayers is the main determinant of the conduction velocity and that the degree of depolarization is different in the various conditions (Fig. 4).

### Cell Characterization and Gap Junctions

**Figure 6:**
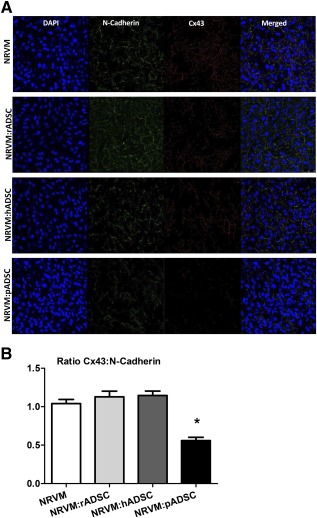
Immunofluorescence micrographs of the various cultures stained with N‐cadherin and Cx43. **(A):** Monolayers of NRVM and monolayers of NRVM cocultured with rADSCs, hADSCs, or pADSC are stained for N‐cadherin and Cx43 (original magnification, ×40). **(B):** The Cx43:N‐cadherin ratio in the various cultures, determined by the number of pixels. Ratios (mean ± SEM) are based on 5–10 images taken in each of three independent experiments. ∗, *p* < .001. Abbreviations: ADSC, adipose cell‐derived stromal cell; DAPI, 4′,6‐diamidino‐2‐phenylindoleh; NRVM, neonatal rat ventricular myocyte; pADSC, pig adipose cell‐derived stromal cell; rADSC, rat adipose cell‐derived stromal cell.

Confluent monolayers of NRVM and cocultures were visualized with light microscopy and immunostaining at day 6. Immunofluorescence staining was performed by using the cardiomyocyte and ADSC markers, α‐actinin and CD44, respectively ( supplemental online Fig. 3). Fluorescent microscopy results revealed that ADSCs were scattered heterogeneously throughout the NRVM monolayer ( supplemental online Fig. 3). Immunofluorescence was performed to visualize connexin 43 (Cx43) and connexin 45 (Cx45) on cardiomyocytes or ADSCs. Cocultures were stained for CD44 and the connexins Cx43 ( supplemental online Fig. 4.1A–4.1D) and Cx45 ( supplemental online Fig. 4.2A–4.2D). In monolayers of NRVM Cx43 and Cx45 are abundantly present ( supplemental online Fig. 4.1A, 4.2A). In monolayers of NRVM cocultured together with rADSC or hADSC Cx43 and Cx45 are also seen ( supplemental online Fig. 4, white arrowheads). However, in the monolayers of NRVM cocultured with pADSC Cx43 and Cx45 are rarely seen ( supplemental online Fig. 4.1D, 4.2D). To quantify these observations, immunofluorescence was performed for N‐cadherin and Cx43 (Fig. 6A) and the ratio of Cx43:N‐cadherin was quantified (Fig. 6B). NRVM monolayers cultured with pADSCs demonstrated significantly lower levels of Cx43:N‐cadherin ratio than control monolayers (0.56 ± 0.04 [± SEM] vs. 1.04 ± 0.05; *p* < .001) and than monolayers of NRVM cultured with rADSCs or hADSCs, respectively (1.12 ± 0.08 and 1.15 ± 0.06; *p* < .001) (Fig. 6B).

## Discussion

In this study we have shown that application of ADSCs, regardless of the species origin, causes heterogeneous conduction slowing in NRVM monolayers. The conduction effect could be attributed to electrotonic interaction and/or paracrine mechanisms. To distinguish between these mechanisms, we first investigated the effects of conditioned medium obtained from the various cocultures. Only conditioned medium from cocultures of NRVMs and pADSCs replicated the effects observed in the cocultures. This indicates the involvement of soluble factors and possible paracrine cross‐talk between the two cell types, in a deleterious way. In humans and rats, the paracrine effects could not be replicated, suggesting that electrotonic coupling plays a more prominent role in these species.

The existence of paracrine cross‐talk between cardiomyocytes and noncardiomyocytes has been suggested by Pedrotty et al. 28 and others 7, 29. Pedrotty et al. demonstrated that conditioned medium from a culture of cardiac fibroblasts altered electrophysiological properties of NRVMs. However, when the same fibroblasts were grown in the presence of NRVMs and the resulting conditioned medium was used, all arrhythmogenic effects disappeared, suggesting that cardiomyocytes were “activated” to produce protective factors that protect them from damaging soluble factors secreted by the fibroblasts 28. To determine whether the observed heterogeneous conduction slowing could be attributed to paracrine cross‐talk between NRVMs and pADSCs or solely to the soluble factors of pADSCs, we used transwell inserts. In these cultures, pADSCs and NRVMs are unable to physically connect, eliminating electrotonic interactions but allowing the exchange of soluble factors. Conditioned media from transwell conditions were used to culture NRVM monolayers, and the results were compared with those obtained in conditioned medium from pADSCs only.

Our results show that ADSCs produced adverse soluble factors that slow the conduction velocity of NRVM monolayers. However, because the conduction slowing by Cme pADSC (16.3 ± 2.4 cm/second) and Cme transwell pADSC (14.6 ± 1.6 cm/second) is less outspoken than in Figure 1 (coculture NRVM:pADSC, *p* < .001 and *p* < .01, respectively) and Figure 2 (Cme NRVM:pADSC, *p* < .01 and *p* < .001, respectively), we deduce that the physical interaction between pADSCs and NRVMs is a prerequisite for this fully paracrine effect. The fact that heterogeneity is not altered with the Cme pADSC whereas the conduction velocity is significantly changed may be related to a different sensitivity to change in uncoupling, resting membrane potential and/or capacitance. We surmise that Cme transwell pADSC influences the interaction in a more severe manner that does Cme pADSC alone. In Cme transwell pADSC and NRVM:pADSC, the cells have had a chance to influence each other and therefore the composition of the conditioned medium is likely to differ from that of the Cme pADSC. Therefore, the communication between pADSCs and NRVMs is necessary for both CV reduction and increased heterogeneity.

In the interaction between NRVMs and ADSCs derived from human and rat, electrotonic coupling likely plays a role. First, condition medium obtained from the coculture of NRVMs and rat or human ADSCs did not replicate the results from the corresponding coculture, and the physical presence of the ADSCs is therefore required for the production of conduction slowing. However, conditioned medium of NRVM:rADSC and NRVM:hADSC cocultures did induce depolarization in NRVM monolayers that was not different from the depolarization in the cocultures. This suggests that soluble factors are responsible for the depolarization but that this is not sufficient for conduction slowing. The relation between RMP and conduction velocity is nonlinear 26, 27, and it is possible that the depolarization induced by conditioned medium of rADSC and hADSC cocultures was slightly less than that in the conditioned medium of pADSC cocultures and therefore insufficient to lead to a conduction slowing. Therefore, it is more probable that the depolarization induced by the soluble factors alone is not enough to induce the heterogeneous conduction slowing in rat and human ADSC cocultures and that additional intercellular coupling is required. The additional intercellular coupling between the rat/human ADSCs and the myocytes would then provide additional depolarization 9, may lead to additional capacitative loading of the NRVM 30, or cause interference with sodium channel function. We have also shown that the relationship between the RMP and the conduction velocity does not deviate from the theoretical sigmoid relation and that it is the same in cocultures and in monolayers subjected to Cme alone (Fig. 5). This suggests that the degree of depolarization determines conduction velocity in each condition. Whether the RMP is determined by paracrine factors or by electrotonic coupling depends on species and conditions.

From the immunofluorescence data, we deduce that connexins (Cx43 and Cx45) are present at the interface between NRVMs and ADSCs in the cocultures with rADSCs and hADSCs. The Cx43:N‐cadherin ratio in these cocultures is not different from that in the control. In these cocultures, electrotonic interaction is therefore possible, although we cannot exclude that the connexins are not entirely electrophysiologically functional (this would require double voltage clamp, which is not feasible in a coculture). Taken together, these data support the idea that electrotonic interaction is the main contributor of the significant heterogeneous conduction slowing in cocultures with rADSCs and hADSCs. This is supported by the observation that Cme of rADSCs and hADSCs were not effective. In contrast, connexins are barely present, and the Cx43:N‐cadherin ratio is significantly lower in cocultures with pADSCs. We demonstrated that the electrophysiological effects of pADSCs are caused through paracrine mechanisms because they are also present in monolayers cultured in Cme NRVM:pADSC. The loss of the connexins can also increase the axial resistance, which is important for the propagation of the cardiac impulse.

**Figure 7:**
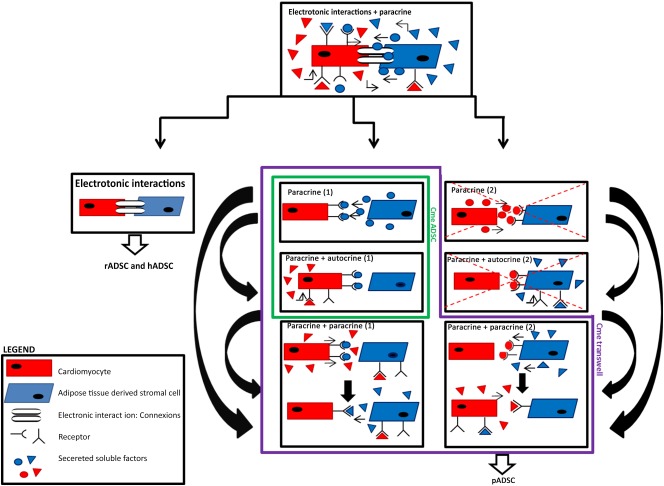
Schematic illustration of the various interactions between neonatal rat ventricular myocyte (NRVM) and ADSC. The figure summarizes the study. First, cocultures of cardiomyocytes and ADSCs are studied. In this scenario, all situations are possible: electrotonic interactions and the various paracrine interactions. We can therefore not exclude or identify which of the situations explains the heterogeneous conduction slowing. The next step is to distinguish between electrotonic interactions and the paracrine interactions. Experiments with conditioned medium (Cme) transwell conditions can allow simple to complicated cross‐talk situations: paracrine only, paracrine + autocrine, or paracrine + paracrine, wherein soluble factors of one cells leads to the secretion of soluble factors by the other cells. This, in turn, stimulates the first cell to secrete different soluble factors. Experiments done with Cme ADSCs can only be explained by paracrine 1 effects of ADSCs on NRVMs, or paracrine factors from ADSCs initiate NRVMs to secrete soluble factors that have an autocrine effect (paracrine + autocrine 1). The situations that are crossed out can also occur; however, the focus of the study is on the effects of ADSC on NRVM conduction properties and not on the effects of NRVM on ADSC. Therefore, these situations are omitted. If we follow the logic of the scheme, we can conclude that the primary mechanism for hADSCs and rADSCs is electrotonic because heterogeneous conduction slowing is not observed when Cme ADSCs are used. When Cme ADSC and Cme transwell of pADSC are used, we still observe heterogeneous conduction slowing, suggesting that the primary effect is paracrine based. Abbreviations: ADSC, adipose cell‐derived stromal cell; hADSC, human adipose cell‐derived stromal cell; pADSC, pig adipose cell‐derived stromal cell; rADSC, rat adipose cell‐derived stromal cell.

All cells have a wide secretome of soluble factors that are secreted and that can influence the behavior and the secretome of other cells. However, these soluble factors in turn can be influenced by environmental factors as well as other soluble factors secreted by other cells or indirectly by the cell its self (autocrine). The exact nature of the soluble factor(s) responsible for inducing the observed heterogeneous conduction slowing is unlikely to be identified and is outside the scope of this paper. Figure 7 provides a schematic summary of this study and the possible cross‐talk interactions that can take place between cardiomyocytes and the various ADSC used.

Our findings that ADSC influence electrophysiological properties of NRVM corroborate those of previous studies, both in vitro and in vivo, that demonstrated that stem cells influence electrophysiological properties 5, 6, 31, 32. However, in this study we specifically studied three different species sources of ADSC: rat, human, and swine. Human and porcine ADSCs were chosen to investigate their arrhythmogenic potential and to see whether porcine ADSCs react differently than human ADSCs. rADSCs were chosen to model allogeneic stem cell application. We have shown that cells of the same species as that of the monolayer cause similar conduction slowing as xenogeneic stem cells.

The study had some limitations. Culturing the two cell types may introduce heterogeneous depolarization of the resting membrane by (a) coupling between ADSCs and NRVMs or (b) paracrine depolarization. Regarding coupling, two potential mechanisms are operative: (a) ADSCs have a less negative RMP than do NRVMs, and coupling may cause depolarization of the NRVMs and (b) coupling may induce a capacitative coupling between the cells that will impede transmission of a propagated impulse. Although we cannot entirely discriminate between the mechanisms, we have addressed the main determinants of CV in our approach. Although repolarization changes may well affect conduction velocity (if the stimulus coincides with the end of the repolarization, as with short premature stimuli), the influence of repolarization abnormalities can be excluded because we did not apply short coupled stimuli.

Although NRVMs and human cardiomyocytes differ, the use of NRVMs (cultured on MEAs) has been established as a reliable model for electrophysiological studies 33 34 35. Compared with adult models, the RMP and CV values obtained in this and other studies are rather low 7, 36, 37. In NRVM monolayers, therefore, sodium channels are partially inactivated. In view of these and our own observations, we assume that propagation in the NRVM monolayers subjected to ADSCs is (also because of depolarization) partially carried by the calcium current, resulting in relatively low conduction velocities. The advantage of the in vitro model of ADSC transplantation is that it allows a controlled application of stromal cell number and conditioned medium to a two‐dimensional model excluding the influence of confounding factors.

## Conclusion

Our results show that ADSCs cause heterogeneous conduction slowing when cocultured on a monolayer of NRVM. Paracrine modulation and intercellular coupling between these two cell types contribute to the formation of a potentially proarrhythmic substrate. We have generated a paracrine‐based proarrhythmic cell model with pADSCs and an electrotonic‐based proarrhythmic cell model with hADSCs and rADSCs. The study shows that adipose stromal cells from different species may interfere with host cardiomyocytes via different mechanisms. We have also demonstrated that the arrhythmic potential of stem cells is maintained even when cross‐species transplantation is used.

Our study was designed to address potential adverse electrophysiological effects of ADSC‐based therapies. Although the question of whether the excreted soluble factors are “beneficial” (e.g., the potential hemodynamic benefit seen in a more clinical setting) is outside the scope of this paper, we have shown that conditioned medium from hADSCs alone does not cause conduction slowing and could thus potentially be used for the possible beneficial soluble factors it contains without having the adverse effects of the interactions these cells can form with cardiomyocytes.

## Author Contributions

J.N.t.S., N.W.S., and M.P.: conception and design, provision of study material, collection and/or assembly of data, data analysis and interpretation, manuscript writing, final approval of manuscript; S.C.M.v.A.: administrative support, provision of study material, collection and/or assembly of data, final approval of manuscript; J.A.P.: administrative support, provision of study material, final approval of manuscript; P.F.H.M.v.D.: conception and design, data analysis and interpretation, final approval of manuscript; J.M.T.d.B.: conception and design, financial support, collection and/or assembly of data, data analysis and interpretation, manuscript writing, final approval of manuscript; M.C.H.: conception and design, financial support, data analysis and interpretation, manuscript writing, final approval of manuscript; R.C.: conception and design, financial support, data analysis and interpretation, manuscript writing, final approval of manuscript.

## Disclosure of Potential Conflicts of Interest

R.C. has a grant from the LeDucq Foundation. The other authors indicated no potential conflicts of interest.

## Supporting information

Supporting Information
