# Supplementary material for: Differential Mechanisms of Myocardial Conduction Slowing by Adipose Tissue‐Derived Stromal Cells Derived from Different Species
Source: Stem Cells Transl Med. 2016 Aug 2;6(1):22–30. doi: 10.5966/sctm.2015-0415 (PMC5442737; doi:10.5966/sctm.2015-0415)
Supplement: Supplementary file 1 — Supporting Information [file SCT3-6-022-s001.pdf]

SUPPLEMENTARY MATERIAL

SUPPLEMENT

Methods

Isolation and culturing of neonatal rat ventricular myocytes

Neonatal rat ventricular myocytes (NRVM) were isolated as follows. One to two days old Wistar rats were decapitated and the hearts were rapidly explanted. Atrial tissue was removed and ventricles were dissected into pieces and left to rotate overnight at 4 °C in HBSS (Gibco, Den Haag, Netherlands) containing trypsin (1 mg/mL; Becton Dickinson BV, Breda, The Netherlands). The following day the enzymatic effect of trypsin was inactivated with culture medium (M199 medium; Gibco; supplemented with 10% heat inactivated fetal bovine serum (FBS; Gibco), 1% HEPES (Gibco #`5630-0-80), 5000 U/L penicillin-G (Sigma,#P7794), 2 mg/L vitamin B12 (Sigma, #V2876), 3,5 g/L glucose, 1% non-essential amino acids (Gibco, #11140-050), and 1% L-glutamine (Gibco,#25030-081)), ventricles were enzymatically dissociated in HBSS containing collagenase type 2 (1 mg/mL, Worthington Vollenhove, The Netherlands, 230 units/mg) at 37 °C, centrifuged at 160 g, 5 minutes and cells were re-suspended in culture medium. To separate fibroblasts from cardiomyocytes, cells were pre-plated in a polystyrene treated T175 cell culture flask at 37°C in 5% $CO_{2,}$ 95% humidity and 21% O_2. After two hours, non-adherent cells, i.e. predominantly NRVM, were collected and were seeded at 1.4 x 10^5/cm^2 onto microelectrode arrays (MEAs; Multi Channel Systems MCS GmbH, Reutlingen, Germany). This array has 60 integrated extracellular electrodes aligned in an 8 by 8 matrix at interelectrode distances of 0.7 mm. MEAs were coated with fibronectin (125 µg/ml BD Biosciences, Breda, The Netherlands) at least two hours prior to NRVM seeding. NRVM were cultured at 37 °C 5% CO_2, 95% humidity and 21% O_2 in culture medium, which was switched to 2% FBS two days after cells were seeded on the MEA. The day after seeding, NRVM were washed twice with HBSS (Gibco) and fresh culture medium was added. Light microscopy was used to determine if

a confluent monolayer had formed in each of the cultures.

Isolation and culture of adipose tissue-derived stromal cells

ADSC were isolated and cultured as described previously (1). Adipose tissue was dissected from rats' inguinal fat (male, Wistar, 7-8 months), porcine (male, 3-4 months old, kindly provided by the department of experimental surgery of the AMC) or human subcutaneous abdominal fat (donated by healthy patients with body mass index below 30 ;Bergman Clinics, The Netherlands) and stored at 4 ºC. Within 24 hours, the adipose tissue was minced and washed extensively with PBS. The tissue fragments were incubated in an equal volume of PBS with 0.1% Collagenase A (Roche Diagnostics, Mannheim, Germany), containing 1% bovine serum albumin (BSA; Sigma-Aldrich, Boston, MA) at 37 °C for 1 hour while exposed to continuous shaking. The enzymatic activity of collagenase was stopped by adding PBS, 1% BSA and digested tissue were filtered through 70 μm filters. The collected cell suspension was subjected and centrifuged at 600xg for 10 min. The supernatant was discarded and stromal vascular fraction (SVF) was incubated with an erythrocyte lysis buffer at 4°C for 5 min. Then, SVF pellet was collected by additional centrifugation. Cells were suspended in culture medium that consisted of DMEM (Lonza Biowhittaker, Verviers, Belgium), supplemented with 10% FBS (Thermo Scientific, Hemel Hempstead, UK), 100 U/mL penicillin, 100 mg/mL streptomycin (Gibco, Invitrogen, Carlsbad, CA) and 2 mM L-glutamine (Lonza Biowhittaker,Verviers, Belgium). The ADSC were seeded at a density of 4 x10^{4} cells/cm^{2} and cultured at 37 ^{o}C, 5% CO_2, 95% humidity and 21% O_2. Culture medium was refreshed every two days till 80-90% confluence was reached. ADSC were propagated at a 1:2 ratio and used from passage 3 onwards for the experiments. Cells were referred to either rat ADSC (rADSC) or human ADSC (hADSC) or pig ADSC (pADSC).

For conditioned medium; confluent flasks of ADSC were cultured in NRVM culture medium containing 2%, after twenty-four hours medium was collected, filtered through a 0.22 µm filter (MILLEX®GV SLGV033RS) labelled Cme-ADSC and stored at -20 ˚C until use.

Experimental conditions

Monolayers of NRVM containing ADSC were prepared by treating ADSC with mitomycin-C (Sigma M4287-2MG) and were labeled with CDFA-SE (Invitrogen Vybrand® CFDA SE Cell Tracer Kit) according to manufactures protocol. ADSC were collected using accutase (Gibco, A11105-01 StemPro®Accutase®), and centrifuged for 5 minutes at 160g. Supernatant was removed and the pellet was re-suspended in NRVM culture medium containing 2% FBS. ADSC were then added to monolayers of NRVM in cell ratios of NRVM:ADSC 1:1. Monolayers of NRVM serving as controls received fresh NRVM culture medium containing 2% FBS. Two days later electrical mapping was performed.

Medium from NRVM monolayers and the monolayers containing the different species of ADSC was collected after two days, filtered through a 0.22 µm filter (MILLEX®GV SLGV033RS) and stored at -20 ˚C until use. This medium was referred to as conditioned medium (Cme) and labeled as follows; Cme NRVM, and Cme NRVM:ADSC.

For the transwell setup monolayers were created in fibronectin coated T12 wells, as described above. On day 4, transwell inserts were placed inside the well and ADSC were seeded into these inserts. The ratio of NRVM:ADSC was 1:2 as the surface area of the inserts is smaller than the T12 wells. Two days later medium was collected, filtered, labelled Cme transwell ADSC and stored at -20 ˚C until used.

In the experiments investigating the paracrine effects, conditioned medium was added to monolayers of NRVM only, two days prior to electrical mapping.

Electrical mapping and microelectrode measurements

The MEAs containing the different cultures were positioned in a temperature controlled (37 ^{o}C MEA holder (TC01/02 Multichannel Systems MSC GmbH). Each MEA harbored 60 electrodes which had terminals in the core portion of the MEA (Supplemental Fig.1). On every day of experimentation two monolayers of NRVM from the same cell isolation served as controls. All cultures were stimulated from at least two stimulation sites using a bipolar extracellular stimulus electrode (twice diastolic stimulation threshold, 1 ms or 2 ms rectangular current pulses). Unipolar electrograms were recorded with a 256-channel amplifier (BioSemi, ActiveTwo, Amsterdam, The Netherlands, 24 bit dynamic range, 122.07 nV LSB, total noise 0.5 µV). Signals were recorded with a sampling frequency of 2048 Hz (filter setting of the amplifiers DC – 400 Hz (- 3dB point). The recordings were made with respect to the integrated reference electrode of the MEA. Conduction velocity (CV) was determined from activation maps constructed using the maximum negative dV/dt as activation time (AT; relative to the time of earliest activation) with the use of a custom made program (2) based on MATLAB R2006b (The MathWorks, Inc., Natick, MA, USA). CV was determined along lines perpendicular to isochronal lines by dividing the distance by the difference in local activation time. Lines had a length of at least 4 electrode distances. Local activation times in the figures are color coded in classes of 5 ms. CV was determined at a basic cycle length (BCL) of 600 ms or during spontaneous activity if pacing was not possible. Based on the method described by Lammers et al. (3) we quantified the heterogeneity in conduction as a measure of arrhythmia vulnerability. Maximum AT differences between each adjacent electrode quartet in the grid were obtained and the total range of maximal AT differences was plotted in a histogram.

Microelectrodes were pulled from glass capillaries (Harvard apparatus GC100F-10) and filled with 3 M KCl. An AgCl covered silver wire was used as a reference electrode. Following activation mapping, action potentials were recorded during pacing at BCL 600 ms. Resting membrane potential (RMP) was taken as the highest negative membrane potential recorded, upstroke velocity was taken as dV/dt max.

Immunostaining and fluorescence imaging

For immunofluorescence, cells (1.25 x 10^5 cells/cm^2) were plated in 12-well plates (MP Biomedicals) containing fibronectin-coated (125 µg/ml BD Biosciences) coverslips and cultured under the same conditions as cells on MEAs.. Separate cultures were made as immunofluorescence could not be performed on MEAs. On the day of the electrophysiological recordings these cultures were fixed with 4% PFA for 10 minutes, permeabilized with 0.1% Triton X-100, and blocked with 1% BSA (Roche BSA fraction V #10735094001). Cells were stained with primary antibodies (mouse anti-sarcomeric-actinin primary antibody; Sigma 1:1000), mouse anti-human monoclonal CD44 primary antibody (Lifespan Biosciences, LS-B1862; 1:250), rabbit anti Connexin 43 (Invitrogen 574366A; 1:200), rabbit anti Connexin 45 (Santa Cruz Biotechnology, sc-25716, 1:100) and mouse anti N-Cadherin (Sigma C2542, 1:100) in 1% BSA overnight at room temperature, washed three times with PBS, and then incubated with secondary antibodies (Alexa Fluor-647 goat anti-mouse IgG (Life Technology, A21235; 1:250), Alexa Fluor-488 goat anti-mouse/rabbit IgG (Life Technology, A11008/A21222; 1:250), for two hours in 1% BSA. Cover-slips were washed an additional three times in PBS and incubated for an additional 10 minutes with either cyto®orange (Life Technology, S11368, 1:1000) or DAPI (Sigma, D9542, 1:40000), washed again with PBS and embedded in 50% glycerol/50% PBS. Examination was performed by Leica SPE confocal laser scanning and Leica Application Suite Advanced Fluorescene (LAS AF) software.

The images obtained from cultures stained for N-Cadherin and Cx43 were analyzed using Image J. Five to ten images were taken in each of three independent experiments, Image J was used to determine the number of pixels for either the Cx43 or N-cadherin channel in each image. Based on the number of pixels we than determined the ratio of Cx43: N-cadherin in each image.

References

1. Przybyt E, Krenning G, Brinker MG et al. Adipose stromal cells primed with hypoxia and inflammation enhance cardiomyocyte proliferation rate in vitro through STAT3 and Erk1/2. J Transl Med 2013;11:39.
2. Potse M, Linnenbank AC, Grimbergen CA. Software design for analysis of multichannel intracardial and body surface electrocardiograms. Comput Methods Programs Biomed 2002;69:225-36.
3. Lammers WJ, Schalij MJ, Kirchhof CJ et al. Quantification of spatial inhomogeneity in conduction and initiation of reentrant atrial arrhythmias. Am J Physiol 1990;259:H1254-63.

Supplemental Figures

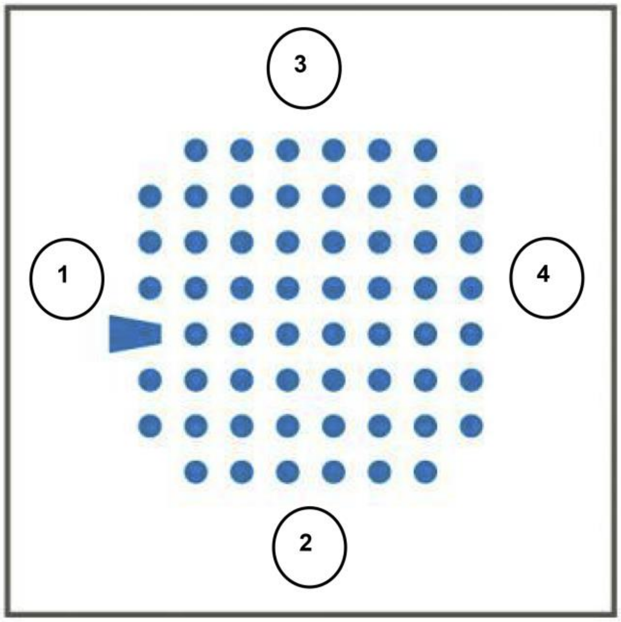

Figure S1. Layout of the 60 electrodes in the MEA. Each electrode has a diameter of 100 µm and an interelectrode distance of 700 µm. Numbers 1-4 represent stimulation positions.

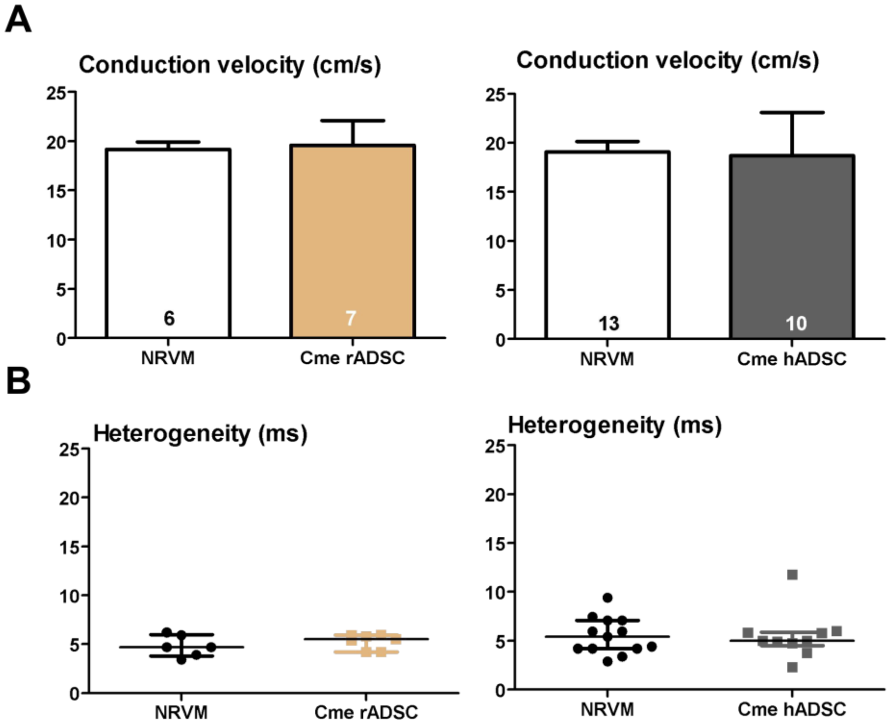

Figure S2. Effect of Cme rADSC and Cme hADSC on monolayers of NRVM. Bar graphs illustrating the effects on **A:** conduction velocity and **B:** conduction heterogeneity in monolayers of NRVM cultured in condition medium obtained from rADSC and hADSC cultures.

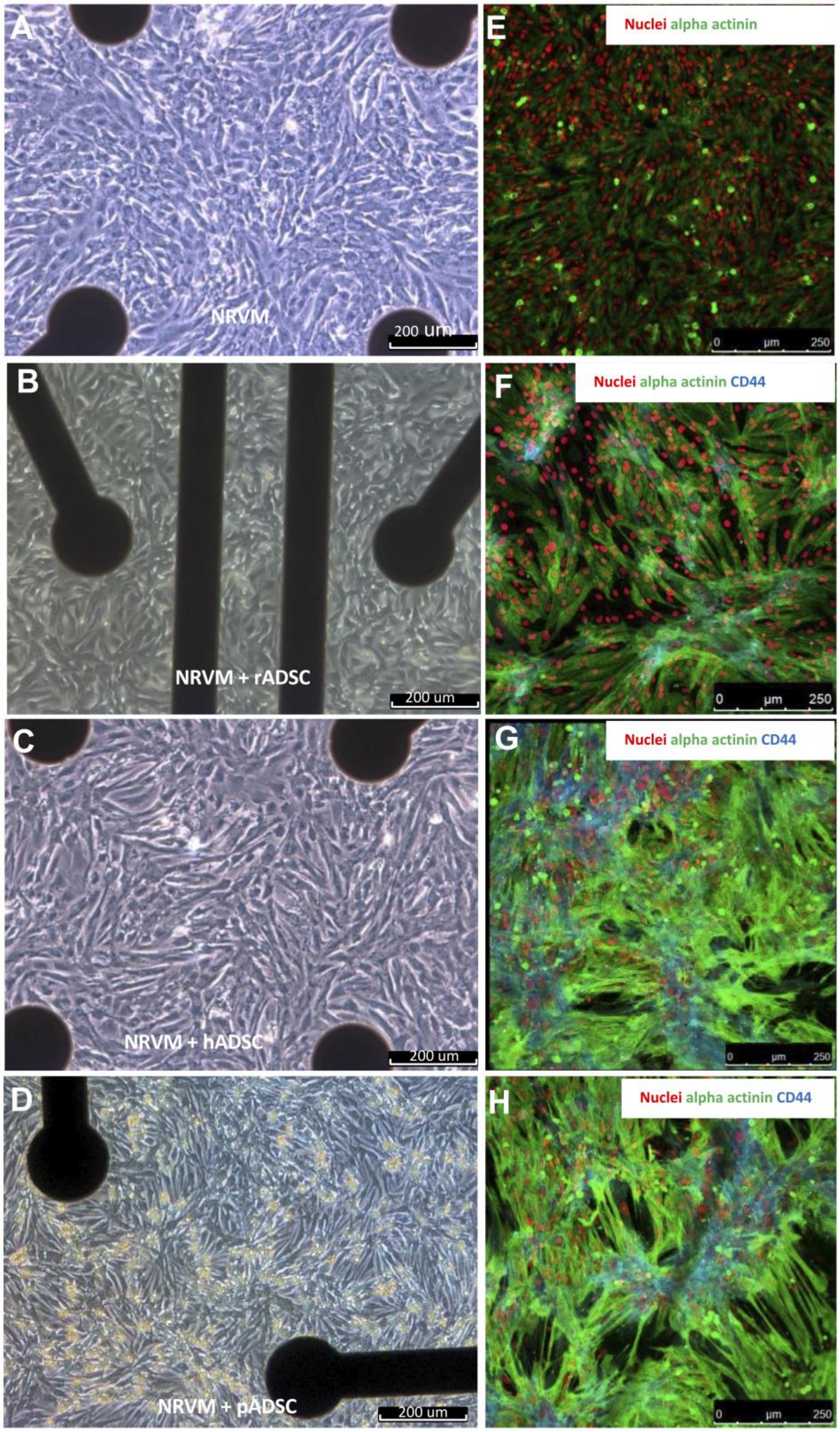
A
NRVM
200 um
E
Nuclei alpha actinin
0 μm 250
B
NRVM + rADSC
200 um
F
Nuclei alpha actinin CD44
0 μm 250
C
NRVM + hADSC
200 um
G
Nuclei alpha actinin CD44
0 μm 250
D
NRVM + pADSC
200 um
H
Nuclei alpha actinin CD44
0 μm 250

FigureS 3. Micrographs of the various cultures. Transmitted light and immune-fluorescent micrographs. **A –D**: Transmitted light micrographs of NRVM monolayer and NRVM monolayers co-cultured with the different ADSC. Black dots and lines are the electrodes in the MEA. **E-H:** Immune-fluorescent micrographs of NRVM monolayer and NRVM monolayers co-cultured with the different ADSC. Please note that these are not the same monolayers as panels A-D. Scale bars located at the bottom right of each image indicate size in µm.

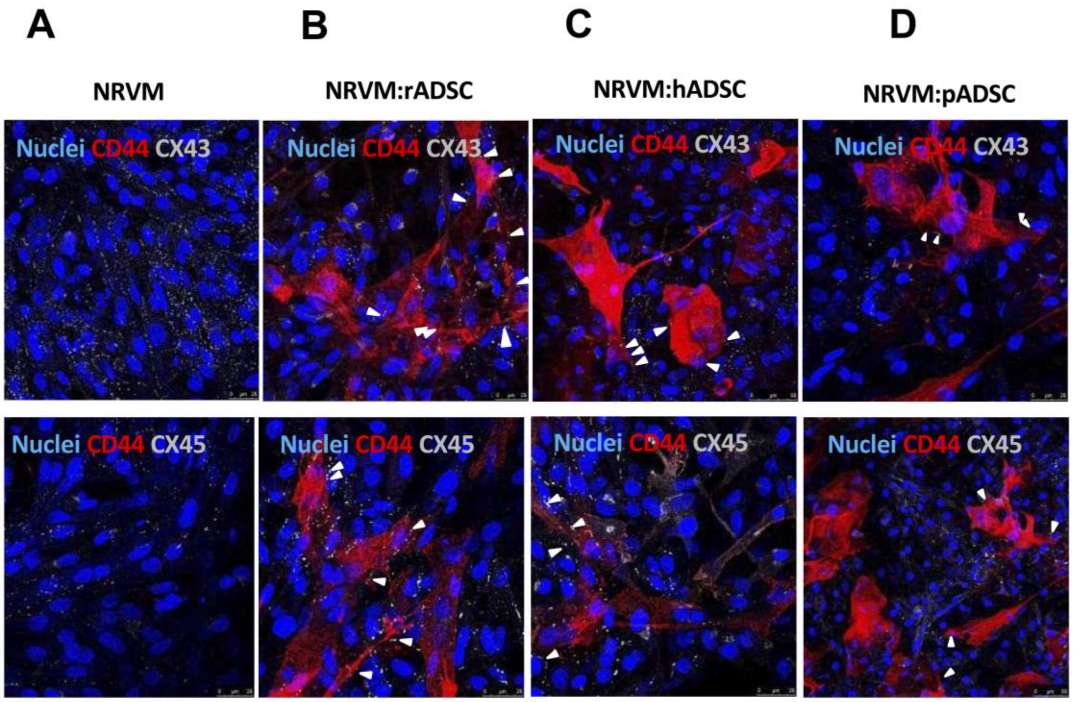

Figure S4. Immunofluorescence micrographs of the various cultures stained with CD44 and Cx43 and Cx45. Monolayers of NRVM are stained with CD44 and Cx43 (**A1**) and with CD44 and Cx45 (**A2**). Monolayers of NRVM co-cultured with rADSC (**B1+B2**), NRVM monolayers co-cultured with hADSC (**C1+C2**) or pADSC (**D1+D2**) are stained for the same markers. Scale bars located at the bottom right of each image indicate size in µm.
